# A cross-sectional comparison of five large language models in answering Chinese-language patient queries on visual field defects associated with brain diseases

**DOI:** 10.3389/fphys.2026.1993774

**Published:** 2026-10-23

**Authors:** Shenghe Li, Ling Wang, Xiaoyan Wang, Ligang Jiang, Weihua Yang, Lu Xie

**Affiliations:** 1 The Second School of Clinical Medicine, Southern Medical University, Guangzhou, China; 2 School of Medical Technology and Engineering, Fujian Medical University, Fuzhou, China; 3 Department of Ophthalmology, Quzhou People’s Hospital, The Quzhou Affiliated Hospital, Wenzhou Medical University, Quzhou, China; 4 Shenzhen Eye Hospital, Shenzhen Eye Medical Center, Southern Medical University, Shenzhen, China

**Keywords:** brain disease, health education, large language model, patient education, quality assessment, visual field defect

## Abstract

**Background:**

Visual field defects associated with brain diseases may arise from stroke, intracranial space-occupying lesions, and other central visual pathway disorders. Patient questions often span symptom recognition, explanation of causes, care triage, treatment, and rehabilitation. Large language models may improve access to patient education. However, their accuracy, logic, coherence, safety, and content accessibility have not been compared under controlled conditions in this high-risk, interdisciplinary Chinese-language setting. This study aimed to compare the performance of five selected large language models across multiple dimensions.

**Methods:**

In this cross-sectional study, 30 fixed Chinese-language questions, refined by experts and phrased from a patient perspective, were submitted to ChatGPT 5.6 Sol, DeepSeek V4 Flash, Gemini 3.7 Flash, Qwen 3.8 Max, and Kimi K3 through a unified application programming interface (API) client. Each model generated one response per question in an independent single-turn session, with web retrieval disabled and conversation history cleared. Three raters, blinded to model identity, independently scored 150 randomly presented responses from 1 to 5 for accuracy, logic, coherence, safety, and content accessibility. Response time, word count, non-space character count, sentence count, paragraph count, and table count were also recorded. Models were compared using the Friedman test followed by Bonferroni-adjusted Wilcoxon signed-rank tests. Inter-rater agreement was assessed using Kendall’s W. Correlations were assessed using Spearman analysis with Benjamini-Hochberg false discovery rate correction.

**Results:**

Agreement among the three raters was high across all five dimensions (Kendall’s W = 0.820–0.844; all P < 0.001). The five models differed in accuracy, coherence, and content accessibility (P < 0.001, P < 0.001, and P = 0.001, respectively). No statistically significant between-model differences were observed in logic or safety (P = 0.247 and P = 0.061, respectively). ChatGPT 5.6 Sol had the highest median accuracy score [4.67 (interquartile range, 4.00–5.00)], and Kimi K3 had the highest median coherence score [4.50 (4.00–5.00)]. Both models had a median content accessibility score of 4.67 (4.00–5.00). Response time, response length, and text structure also differed among models. However, their correlations with expert quality scores were generally weak (|ρ| ≤ 0.279).

**Conclusion:**

The five large language models exhibited distinctly multidimensional performance when answering Chinese-language patient questions about visual field defects associated with brain diseases. No single model consistently performed best across all clinically relevant dimensions. These findings are specific to the model versions tested on 15 August 2026; stability across repeated generations was not assessed. Response speed, length, and structure cannot substitute for direct assessment of medical quality. The absence of a statistically significant difference in safety ratings under controlled conditions does not imply equivalent or adequate clinical safety. Large language models are better suited to supporting patient education and clinician-patient communication under clinical supervision than to making diagnostic or treatment decisions independently.

## 1 Introduction

Visual field defects are an important clinical manifestation of injury to the central visual pathway caused by brain diseases. Patients often present with nonspecific complaints such as “blurred vision” or “a missing area” in their field of view. However, lesions may involve the globe, optic nerve, optic chiasm, optic tract, optic radiations, or visual cortex. Injury to the central visual pathway can produce bitemporal or homonymous visual field defects. Such defects occur in stroke, intracranial tumors, traumatic brain injury, neurosurgical procedures, and demyelinating diseases (Donaldson and Margolin, 2021; Newman and Biousse, 2025). They can restrict driving, walking, and obstacle detection. In severe cases, they are also associated with an increased risk of falls requiring hospitalization (Qiu et al., 2014; Manners et al., 2024). These inquiries therefore extend beyond symptom explanation within a single specialty. They raise multidisciplinary questions about differential diagnosis, lesion localization, risk stratification, specialist referral, treatment of the underlying disorder, and visual rehabilitation. Relevant care may involve ophthalmology or neuro-ophthalmology, neurology, neurosurgery, diagnostic imaging, and visual rehabilitation (Wang et al., 2024; Bsteh et al., 2025). The accuracy, safety, and accessibility of these responses may influence how patients judge the urgency of care and their next steps.

Large language models (LLMs) and related artificial intelligence tools are being explored in clinical, educational, and research settings (Thirunavukarasu et al., 2023). Artificial intelligence has also entered ophthalmic clinical education (Yuan et al., 2025). For example, an AI agent has combined image classification with a retrieval-augmented knowledge base to address text and image inquiries about myopia (Wu et al., 2025b). Although LLMs may expand access to medical information, they can also disseminate inaccurate or distorted content (Clusmann et al., 2023). Previous studies of ophthalmic patient questions have identified incomplete information, errors, and potentially harmful advice in model responses. Some responses also require an excessively high reading level and should not directly guide patient decisions without professional supervision (Cappellani et al., 2024; Hamzeh et al., 2026). Performance within the same system may fluctuate over time (Gupta et al., 2025). It also varies across models, versions, and ophthalmic topics (Thirunavukarasu et al., 2024; Wei et al., 2025) and across question types in Chinese medical licensing examinations (Tang et al., 2026). Recent studies have compared multiple models for disease-specific questions from parents of children with congenital cataracts and from patients with diabetic retinopathy (Cang et al., 2026; Jiang et al., 2026). Huang et al. evaluated five LLMs on common ophthalmology questions using five quality dimensions and character, word, and sentence counts (Huang et al., 2025). However, these studies do not establish whether LLMs can appropriately address patient questions about visual field defects associated with brain diseases. In this setting, models must explain visual field symptoms and identify warning signs associated with central causes. They must also provide appropriate cross-specialty guidance and address treatment, rehabilitation, care, psychological support, and prognosis. The present study therefore extends this multidimensional evaluation to consultations about visual field defects associated with brain diseases. It also incorporates response time, paragraph counts, and table counts, and explores associations between output characteristics and expert-rated response quality.

To address this gap, this cross-sectional study compared responses from five API-accessed LLMs: ChatGPT 5.6 Sol, DeepSeek V4 Flash, Gemini 3.7 Flash, Qwen 3.8 Max, and Kimi K3. Each model answered 30 fixed Chinese-language questions phrased from a patient perspective. The questions covered six domains: symptom recognition and self-assessment; brain diseases and visual-field changes; care-seeking and diagnostic testing; treatment and intervention; rehabilitation and daily care; and psychological support and prognosis. All responses were generated using a standardized, single-turn process without external retrieval. Raters independently assessed accuracy, logic, coherence, safety, and content accessibility using uniform criteria. We also quantified response time and text-structure characteristics to examine their associations with expert-rated response quality. The aim was to identify strengths and risks for this specific task and inform clinically supervised patient education and communication, rather than produce a generalizable ranking of models.

## 2 Materials and methods

### 2.1 Study design and reporting standards

This cross-sectional comparative study evaluated the performance of five selected API-accessible LLM chatbots in answering questions from patients with visual field defects. A previous systematic review of health-advice chatbots found substantial heterogeneity in reporting model versions, query dates, technical para meters, and prompting strategies. Transparent documentation of model configurations and query procedures is therefore essential for interpreting and replicating results (Huo et al., 2025a). Following relevant recommendations from the Chatbot Assessment Reporting Tool (CHART) (Huo et al., 2025b), we prospectively recorded model identifiers, access routes, testing dates, prompting strategies, and evaluation procedures. **Figure 1** summarizes the study design and analysis workflow.

**Figure 1:**
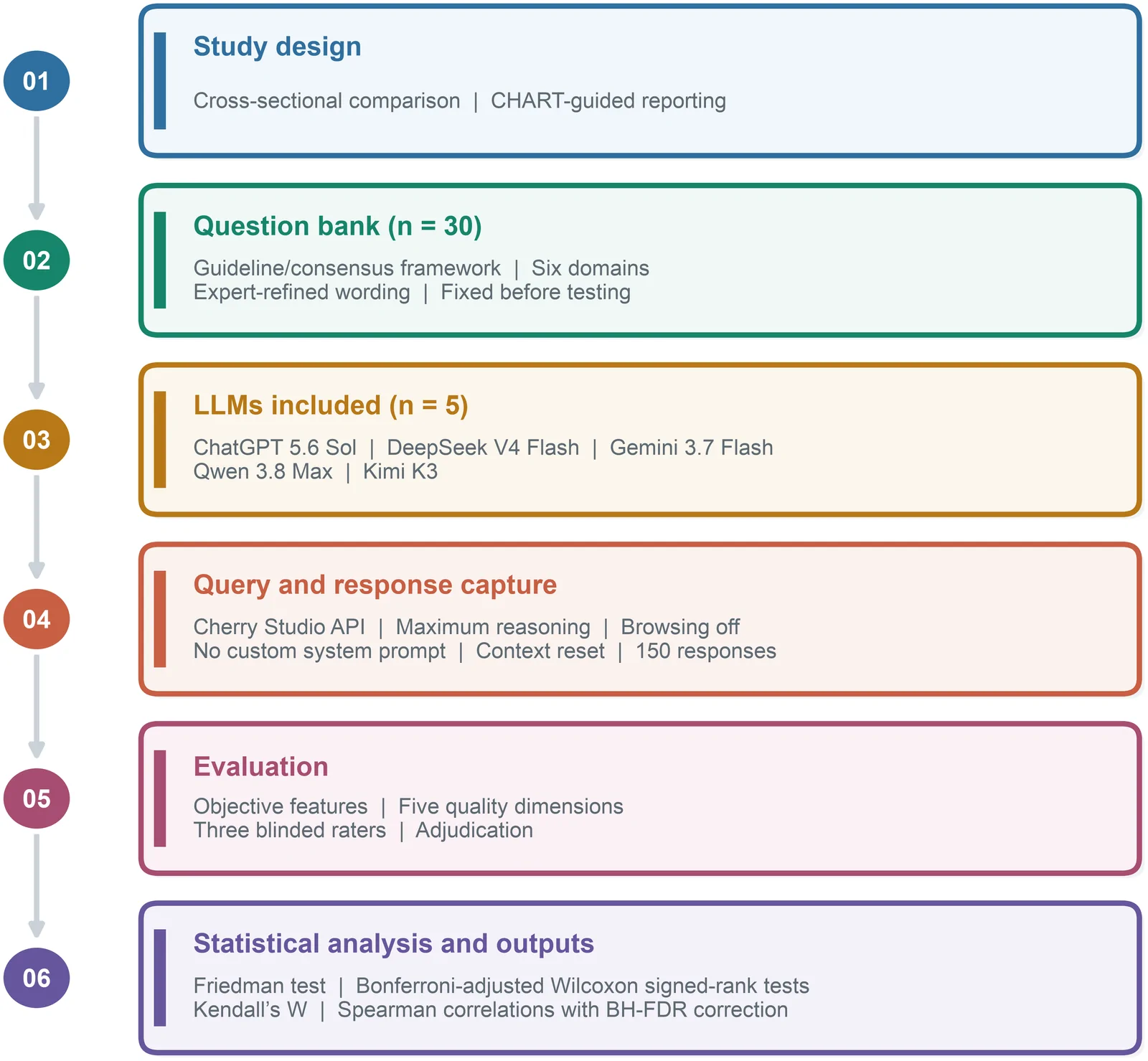
Study design and analysis workflow for evaluating LLM responses to Chinese-language patient questions about visual field defects associated with brain diseases.

### 2.2 Question bank construction

We constructed a bank of 30 fixed Chinese-language questions to evaluate LLM responses to inquiries from patients with visual field defects and their families. The clinical framework was based primarily on four sources: the European Stroke Organisation guideline on visual impairment after stroke; the Pituitary Society international consensus on pituitary incidentaloma; the consensus guideline on managing idiopathic intracranial hypertension; and the American College of Radiology Appropriateness Criteria for vision loss (Mollan et al., 2018; Fleseriu et al., 2025; Friedman et al., 2025; Rowe et al., 2025). These guidelines cover recognition of visual symptoms, evaluation of cerebral and neuro-ophthalmic causes, care-seeking, imaging, visual field assessment, treatment of the underlying disorder, visual rehabilitation, and long-term follow-up. Informed by previous research, question development also drew on common clinical inquiries and patient-education scenarios. Questions were grouped into six predefined categories: symptom recognition and self-assessment; brain diseases and visual-field changes; care-seeking and diagnostic testing; treatment and intervention; rehabilitation and daily care; and psychological support and prognosis (Alqudah et al., 2024).

All questions were discussed and revised by a clinical expert panel. Medical terminology was minimized and wording was adapted to the patient perspective to improve readability and simulate real-world inquiries. The questions were finalized before model testing, and their wording, order, and classification remained unchanged throughout the experiment. For transparency, **Supplementary Table S2** provides the Chinese version, English rendering, and predefined category for each of the 30 questions.

### 2.3 Selection of LLMs

Given the rapid evolution of LLMs, we focused on recent API-accessible models from different developers available at the time of testing. Accordingly, we selected ChatGPT 5.6 Sol, DeepSeek V4 Flash, Gemini 3.7 Flash, Qwen 3.8 Max, and Kimi K3. These models supported Chinese-language dialogue, remained accessible through APIs during testing, and could be evaluated through a common question-input workflow in Cherry Studio. **Table 1** reports the exact API model identifiers and provider-reported release, revision, and knowledge-cutoff information relevant to testing on 15 August 2026. **Supplementary Tables S1A, B** provide API access routes and operating settings.

**Table 1:** Model identity and provider-reported version metadata for API testing on 15 August 2026.

Model (developer)	Exact API model identifier	Release/pre-test model update	Build/snapshot provenance	Provider-reported knowledge cutoff
ChatGPT 5.6 Sol (OpenAI)	gpt-5.6-sol	9 July 2026 (GA)	NV	16 February 2026
DeepSeek V4 Flash (DeepSeek)	deepseek-v4-flash	24 April 2026 (preview); 31 July 2026 (API update)	DeepSeek-V4-Flash-0731 (public label); snapshot NV	NR
Qwen 3.8 Max (Alibaba/Qwen)	qwen3.8-max	3 August 2026 (API release)	NV	NR
Kimi K3 (Moonshot AI)	kimi-k3	16 July 2026 (API release)	NV	NR
Gemini 3.7 Flash (Google)	gemini-3.7-flash	13 August 2026 (API release)	NV	March 2026

GA, general availability; NV, build or snapshot identifier not verified; NR, knowledge cutoff not identified in reviewed official documentation. For Gemini, knowledge in some domains may remain limited to January 2025.

### 2.4 Data collection and extraction

Data were collected on August 15, 2026. Researchers used API keys for the five models through Cherry Studio, an open-source client, to obtain responses to the 30-question visual field defect bank. API access for all five models was purchased through personal accounts. All responses were collected within the specified window of 09:00-18:00 to reduce output instability associated with fluctuations in server load. To minimize the influence of configuration differences on response length, response time, and content, the settings available for each model in Cherry Studio were configured according to a common protocol. At the time of data collection, the reasoning or thinking setting for each model was set to the highest level available for that model in Cherry Studio. Temperature, top-p, and the output token limit were not manually overridden in Cherry Studio. Web search and browsing were disabled, and no custom system prompt was used. Conversation history was cleared before each query, and every response was collected in an independent single-turn session to minimize the influence of prior conversational context. Each of the 30 questions was submitted once to each model, yielding one response per model–question pair and 150 responses in total. The model label used for evaluation, reasoning and generation setting, search/browsing status, system prompt and prompting, session control, query language, and output handling and timing are summarized in **Supplementary Table S1B**.

### 2.5 Evaluation measures

#### 2.5.1 Objective output measures

To compare objective response characteristics across models, we recorded response time and extracted the word, non-space character, sentence, paragraph, and table counts for each response. Response time was measured manually in seconds from query submission until the model completed its response. Manual timing was used instead of the API-reported time to apply a consistent timing procedure across API providers. It was also intended to approximate user-perceived waiting time during model interaction.

The measure labelled “word count” was operationally defined as the sum of the counts of Chinese characters, English words, and numeric characters displayed by Toolsonline (https://toolsonline.run/zh/word-count). Chinese characters were counted individually, without Chinese lexical segmentation. English words followed the website’s word-boundary rules, and numeric characters were counted individually (e.g., “100” contributed three units). For each of the 150 responses, the webpage-displayed component counts were checked individually and summed to obtain the final word-count value. Non-space character count excluded whitespace, including spaces, tabs, and line breaks. Non-space character, sentence, and paragraph counts were obtained using Charliehome (https://wc.charliehome.xx.kg/) and Toolsonline. Results for these three measures were cross-checked between the two platforms.

Tables were counted as identifiable, discrete tables within each response. These measures described output speed, response length, and content organization. They were not treated as substitutes for response quality.

#### 2.5.2 Five-dimensional scoring rubric

The subjective quality of generated responses was assessed using a prespecified five-dimensional rubric covering accuracy, logic, coherence, safety, and content accessibility. Each dimension was rated on an ordinal scale from 1 to 5, with higher scores indicating better performance. The five dimensions were evaluated independently; a low score in one dimension did not automatically reduce scores in others.

Accuracy assessed only whether the medical information was correct. Judgments were based primarily on relevant clinical guidelines, expert consensus statements, and prespecified reference answers, without considering language or organization. Logic assessed whether reasons, conditions, and conclusions supported one another and whether responses contained contradictions, causal confusion, or unsupported conclusions. Coherence assessed the organization, sequencing, and clarity of the response, including whether the main conclusion was prominent, without reassessing medical correctness. Safety assessed whether a response could harm a patient or delay care and whether it provided necessary warnings, danger signs, and timing for seeking care. Content accessibility assessed whether a lay patient could understand the response and use its advice to take appropriate action. This included explanations of medical terminology, clarity of key information, and actionable next steps. Greater length was not assumed to improve accessibility. Ratings were reduced when redundant information obscured the central message. The complete 1–5 scoring anchors for each dimension are provided in **Supplementary Table S3**.

#### 2.5.3 Inter-rater agreement and resolution of disagreements

Three researchers engaged in clinical ophthalmology research scored all 150 responses generated by the five models for the 30 questions. Before formal scoring, researchers removed model names and other information that could identify the source of each response. Responses were presented in random order. The three raters, blinded to model identity, independently assessed all responses using the prespecified five-dimensional rubric and were not permitted to consult one another during scoring.

After independent scoring, an online discussion was convened when the range of ratings for any dimension of a response was at least 2 points. The three raters discussed the disagreement and assessed the response with reference to the prespecified standard answer. If they could not reach a final score, the response was referred to a fourth expert for adjudication. When the range was less than 2 points, no discussion was initiated, and the mean of the three original ratings was used as the final score. Kendall’s W was used to assess agreement among the three raters based only on their original independent ratings before disagreement resolution. All subsequent analyses of the five quality dimensions used the final scores, except the analysis of inter-rater agreement.

### 2.6 Statistical analysis

All statistical analyses except correlation analyses were performed using IBM SPSS Statistics version 32.0.0.0 (build 139; IBM Corp., Armonk, NY, USA). Correlation analyses were performed using R version 4.6.1 (R Foundation for Statistical Computing, Vienna, Austria).

All six objective measures were summarized as means ± standard deviations and medians with the first and third quartiles (Q1, Q3). Final scores for the five quality dimensions were summarized as medians (Q1, Q3). For each model, we additionally reported the number and percentage of responses with final safety scores ≤3 and the subset with scores ≤2. Because all five models answered the same 30 questions, comparisons were paired by question, yielding 30 matched sets of observations for each measure.

Overall differences among models were assessed using the Friedman test for each measure, with Kendall’s W reported as the effect size. Pairwise model comparisons were also performed using two-sided Wilcoxon signed-rank tests, with Bonferroni correction applied to the 10 comparisons within each measure. Pairwise results were reported regardless of the statistical significance of the overall test. Statistical significance was defined as P < 0.05 for overall tests and Bonferroni-adjusted P < 0.05 for pairwise comparisons.

Agreement among the three raters was assessed using Kendall’s W based exclusively on their original independent ratings before disagreement resolution. All other analyses involving the five quality dimensions used the final scores determined according to the procedure described in Section 2.5.3.

Exploratory two-sided Spearman rank correlations were calculated across the 150 pooled responses for the 11 measures comprising six objective characteristics and five quality ratings. The Benjamini–Hochberg procedure was applied to the 55 unique pairwise correlation tests, with adjusted q < 0.05 considered statistically significant. These analyses described exploratory associations in the pooled sample and were not interpreted as within-model or causal relationships.

## 3 Results

### 3.1 Objective characteristics of LLM responses

Response times differed significantly among the five models (Friedman χ²(4) = 37.672, Kendall’s W = 0.314, P < 0.001; **Table 2**; **Figure 2a**). DeepSeek V4 Flash had the shortest mean response time (17.333 ± 7.743 s), followed by Gemini 3.7 Flash (20.367 ± 2.723 s), ChatGPT 5.6 Sol (24.600 ± 14.712 s), Kimi K3 (30.900 ± 17.349 s), and Qwen 3.8 Max (36.067 ± 16.360 s; **Table 2**). Bonferroni-adjusted Wilcoxon signed-rank tests showed that Qwen 3.8 Max responded more slowly than ChatGPT 5.6 Sol (P = 0.004), DeepSeek V4 Flash, and Gemini 3.7 Flash (both P < 0.001; **Table 3**). Kimi K3 also responded more slowly than DeepSeek V4 Flash (P = 0.007) and Gemini 3.7 Flash (P = 0.036; **Table 3**). No other pairwise comparison reached statistical significance (all P ≥ 0.077; **Table 3**).

**Table 2:** Comparison of objective response characteristics across five large language models.

Indicator	Summary	ChatGPT 5.6 Sol	DeepSeek V4 Flash	Qwen 3.8 Max	Kimi K3	Gemini 3.7 Flash	Friedman χ²	Kendall’s W	P value
Response time (s)	Mean ± SD	24.600 ± 14.712	17.333 ± 7.743	36.067 ± 16.360	30.900 ± 17.349	20.367 ± 2.723	37.672	0.314	<0.001
	Median (Q1, Q3)	22.00 (15.75, 27.75)	17.00 (9.00, 24.00)	28.50 (23.00, 52.00)	29.50 (14.75, 38.25)	20.00 (18.75, 22.00)			
Words	Mean ± SD	499.267 ± 210.212	932.933 ± 374.506	1337.567 ± 712.704	598.567 ± 117.341	1108.267 ± 216.414	81.035	0.675	<0.001
	Median (Q1, Q3)	439.00 (340.75, 652.00)	933.00 (638.75, 1164.50)	1001.50 (742.50, 1974.50)	570.50 (529.75, 716.25)	1115.50 (972.00, 1239.50)			
Non-space characters	Mean ± SD	569.267 ± 248.439	1096.200 ± 439.405	1553.633 ± 850.320	703.667 ± 136.676	1326.300 ± 274.542	81.227	0.677	<0.001
	Median (Q1, Q3)	492.00 (392.00, 741.50)	1117.00 (746.25, 1352.00)	1126.00 (857.00, 2295.75)	674.50 (609.50, 809.00)	1340.00 (1169.25, 1507.75)			
Paragraphs	Mean ± SD	6.033 ± 3.068	12.667 ± 4.147	31.567 ± 21.919	13.267 ± 2.406	12.967 ± 3.264	72.694	0.606	<0.001
	Median (Q1, Q3)	6.00 (3.00, 7.50)	12.00 (9.00, 14.25)	22.00 (13.75, 51.25)	13.00 (11.00, 15.00)	13.00 (10.00, 14.25)			
Sentences	Mean ± SD	13.967 ± 6.327	28.400 ± 12.400	53.133 ± 48.347	9.033 ± 5.334	29.333 ± 7.237	87.428	0.729	<0.001
	Median (Q1, Q3)	11.50 (9.00, 18.25)	28.00 (15.75, 41.00)	31.50 (20.75, 75.75)	8.50 (5.00, 12.25)	27.00 (25.00, 33.50)			
Tables	Mean ± SD	0.167 ± 0.379	0.167 ± 0.379	0.267 ± 0.450	0.533 ± 0.571	0.233 ± 0.430	20.195	0.168	<0.001
	Median (Q1, Q3)	0.00 (0.00, 0.00)	0.00 (0.00, 0.00)	0.00 (0.00, 1.00)	0.50 (0.00, 1.00)	0.00 (0.00, 0.25)			

Data are presented as mean ± SD and median (Q1, Q3). Each model answered the same 30 questions (n = 30 responses per model). Overall differences among models were assessed using Friedman tests, with responses paired by question (df = 4). Kendall’s W denotes effect size. SD, sample standard deviation; Q1 and Q3, 25th and 75th percentiles.

**Figure 2:**
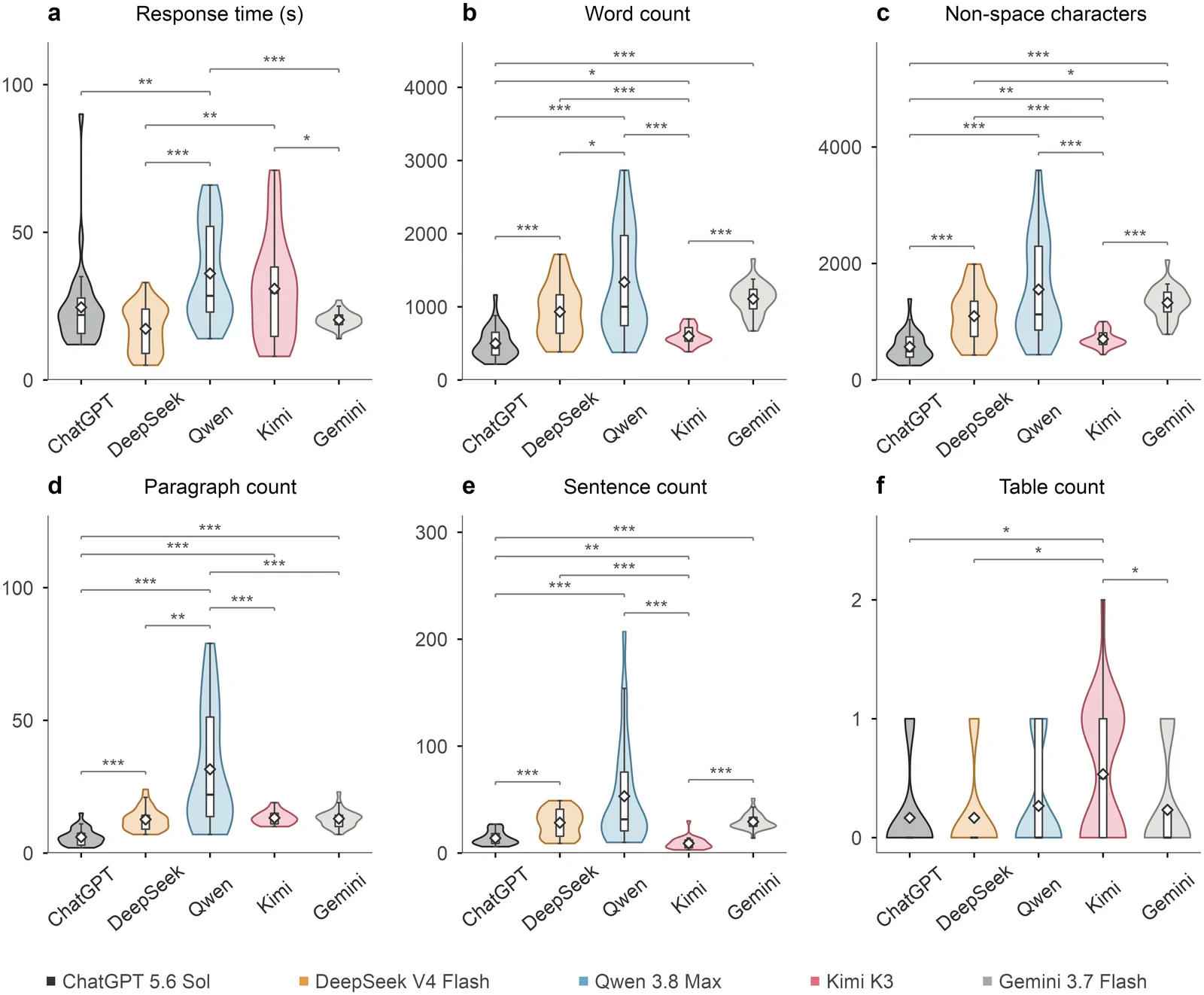
Distributions of response time and objective text characteristics across five large language models. **(a)** Response time (s); **(b)** word count; **(c)** non-space character count; **(d)** paragraph count; **(e)** sentence count; and **(f)** table count. Each model answered the same 30 questions once (n = 30 responses per model). Colored violins show kernel density estimates, trimmed to the observed ranges and scaled to equal maximum widths. White boxes span the first to third quartiles (Q1–Q3), horizontal lines indicate medians, and white diamonds indicate means. Whiskers extend to the most extreme observations within Q1 − 1.5 × IQR and Q3 + 1.5 × IQR, where IQR = Q3 − Q1. All observations contribute to the distributions and summaries; individual data points are not shown. Panel **(f)** shows smoothed distributions of discrete table counts of 0, 1, or 2. Brackets indicate significant pairwise comparisons from two-sided Wilcoxon signed-rank tests, paired by question, with Bonferroni correction for the 10 comparisons within each measure. Asterisks correspond to the adjusted P values reported in **Table 3**: *P < 0.05, **P < 0.01, and ***P < 0.001.

**Table 3:** Pairwise comparisons of objective measures across five large language models.

Comparison	Response time (s)	Words	Non-space characters	Paragraphs	Sentences	Tables
ChatGPT 5.6 Sol vs. DeepSeek V4 Flash	0.077	<0.001	<0.001	<0.001	<0.001	1.000
ChatGPT 5.6 Sol vs. Qwen 3.8 Max	0.004	<0.001	<0.001	<0.001	<0.001	0.833
ChatGPT 5.6 Sol vs. Kimi K3	1.000	0.011	0.002	<0.001	0.009	0.023
ChatGPT 5.6 Sol vs. Gemini 3.7 Flash	0.897	<0.001	<0.001	<0.001	<0.001	1.000
DeepSeek V4 Flash vs. Qwen 3.8 Max	<0.001	0.045	0.073	0.001	0.094	1.000
DeepSeek V4 Flash vs. Kimi K3	0.007	<0.001	<0.001	1.000	<0.001	0.045
DeepSeek V4 Flash vs. Gemini 3.7 Flash	0.503	0.064	0.047	1.000	1.000	1.000
Qwen 3.8 Max vs. Kimi K3	1.000	<0.001	<0.001	<0.001	<0.001	0.325
Qwen 3.8 Max vs. Gemini 3.7 Flash	<0.001	1.000	1.000	<0.001	0.256	1.000
Kimi K3 vs. Gemini 3.7 Flash	0.036	<0.001	<0.001	1.000	<0.001	0.027

Pairwise comparisons between models used Wilcoxon signed-rank tests. The table reports Bonferroni-adjusted P values. An adjusted P value < 0.05 was considered statistically significant.

Word counts differed significantly among the five models (Friedman χ²(4) = 81.035, Kendall’s W = 0.675, P < 0.001; **Table 2**; **Figure 2b**). Qwen 3.8 Max had the highest mean word count (1337.567 ± 712.704), followed by Gemini 3.7 Flash (1108.267 ± 216.414), DeepSeek V4 Flash (932.933 ± 374.506), Kimi K3 (598.567 ± 117.341), and ChatGPT 5.6 Sol (499.267 ± 210.212; **Table 2**). ChatGPT 5.6 Sol generated fewer words than DeepSeek V4 Flash, Qwen 3.8 Max, and Gemini 3.7 Flash (all P < 0.001) and fewer than Kimi K3 (P = 0.011; **Table 3**). Kimi K3 generated fewer words than DeepSeek V4 Flash, Qwen 3.8 Max, and Gemini 3.7 Flash (all P < 0.001; **Table 3**). Qwen 3.8 Max generated more words than DeepSeek V4 Flash (P = 0.045). Differences between Qwen 3.8 Max and Gemini 3.7 Flash (P = 1.000) and between DeepSeek V4 Flash and Gemini 3.7 Flash (P = 0.064) were not statistically significant (**Table 3**).

Results for non-space character counts were generally consistent with those for word counts (Friedman χ²(4) = 81.227, Kendall’s W = 0.677, P < 0.001; **Table 2**; **Figure 2c**). Qwen 3.8 Max had the highest mean count (1553.633 ± 850.320), followed by Gemini 3.7 Flash (1326.300 ± 274.542), DeepSeek V4 Flash (1096.200 ± 439.405), Kimi K3 (703.667 ± 136.676), and ChatGPT 5.6 Sol (569.267 ± 248.439; **Table 2**). Although Qwen 3.8 Max had the highest mean word and non-space character counts, Gemini 3.7 Flash had the highest medians for both measures (**Table 2**). Qwen 3.8 Max, Gemini 3.7 Flash, and DeepSeek V4 Flash each generated more non-space characters than ChatGPT 5.6 Sol and Kimi K3 (all adjusted P < 0.001). Kimi K3 also generated more than ChatGPT 5.6 Sol (adjusted P = 0.002; **Table 3**). Gemini 3.7 Flash generated slightly more non-space characters than DeepSeek V4 Flash (P = 0.047). Differences between Qwen 3.8 Max and DeepSeek V4 Flash and between Qwen 3.8 Max and Gemini 3.7 Flash were not statistically significant (**Table 3**).

The number of paragraphs also differed significantly among models (Friedman χ²(4) = 72.694, Kendall’s W = 0.606, P < 0.001; **Table 2**; **Figure 2d**). Qwen 3.8 Max generated the most paragraphs (31.567 ± 21.919; **Table 2**), significantly more than each of the other four models (all P ≤ 0.001; **Table 3**). ChatGPT 5.6 Sol generated the fewest paragraphs (6.033 ± 3.068; **Table 2**), significantly fewer than each of the other four models (all P < 0.001; **Table 3**). Mean paragraph counts were similar for DeepSeek V4 Flash, Kimi K3, and Gemini 3.7 Flash, with no significant pairwise differences among these models (all P = 1.000; **Table 3**).

Sentence count showed the largest between-model difference among the six objective measures (Friedman χ²(4) = 87.428, Kendall’s W = 0.729, P < 0.001; **Table 2**; **Figure 2e**). Qwen 3.8 Max had the highest mean sentence count (53.133 ± 48.347), with substantial variability across questions. Its median sentence count was 31.50 (Q1, Q3: 20.75, 75.75). Kimi K3 had the lowest mean count (9.033 ± 5.334; **Table 2**). After Bonferroni correction, Kimi K3 generated fewer sentences than ChatGPT 5.6 Sol (P = 0.009), DeepSeek V4 Flash, Qwen 3.8 Max, and Gemini 3.7 Flash (all three P < 0.001; **Table 3**). ChatGPT 5.6 Sol generated fewer sentences (13.967 ± 6.327) than DeepSeek V4 Flash, Qwen 3.8 Max, and Gemini 3.7 Flash (all P < 0.001; **Table 3**). Sentence counts did not differ significantly among DeepSeek V4 Flash, Qwen 3.8 Max, and Gemini 3.7 Flash (all P ≥ 0.094; **Table 3**).

Although the models generated few tables overall, their table-count distributions differed significantly (Friedman χ²(4) = 20.195, Kendall’s W = 0.168, P < 0.001; **Table 2**; **Figure 2f**). Kimi K3 had the highest median table count, at 0.50 (Q1, Q3: 0.00–1.00); all other models had a median of 0 (**Table 2**). Kimi K3 generated more tables than ChatGPT 5.6 Sol (P = 0.023), DeepSeek V4 Flash (P = 0.045), and Gemini 3.7 Flash (P = 0.027). Its difference from Qwen 3.8 Max was not statistically significant (P = 0.325; **Table 3**). All other pairwise comparisons were also nonsignificant (all P ≥ 0.833; **Table 3**). Table count showed the smallest between-model difference among the objective measures (Kendall’s W = 0.168; **Table 2**).

### 3.2 Experts evaluation of response quality

#### 3.2.1 Inter-rater agreement

The three raters showed high agreement across all five evaluation dimensions. Kendall’s W ranged from 0.820 to 0.844: W = 0.833 for accuracy, W = 0.838 for logic, W = 0.839 for coherence, W = 0.820 for safety, and W = 0.844 for content accessibility. All tests were statistically significant (P < 0.001). **Figure 3** presents Kendall’s W for each dimension.

**Figure 3:**
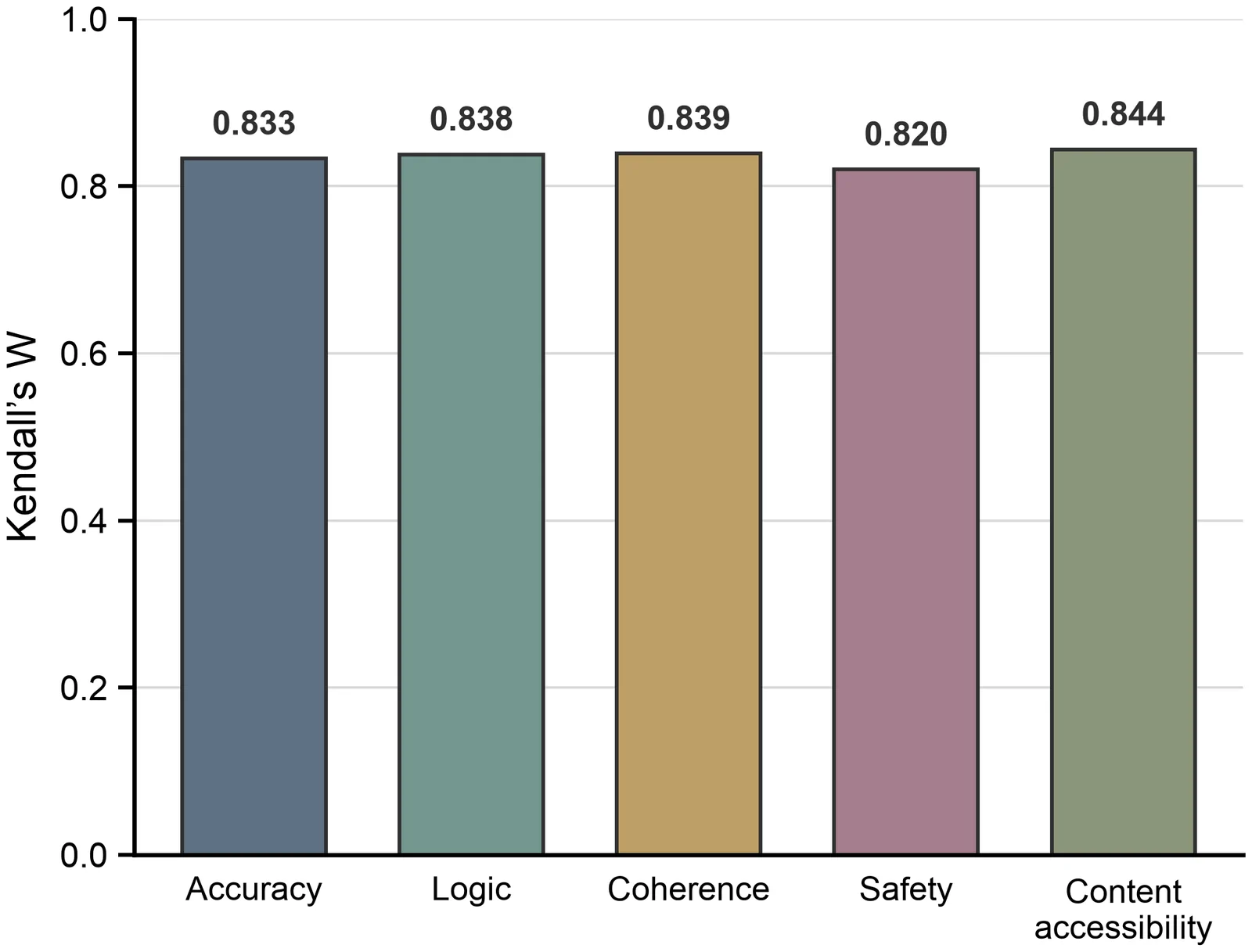
Inter-rater agreement across the five quality dimensions. Kendall’s W was used to assess agreement for ratings of accuracy, logic, coherence, safety, and content accessibility.

#### 3.2.2 Comparison across five quality dimensions

Friedman tests showed significant differences among the five models in accuracy, coherence, and content accessibility. No significant differences were observed in logic or safety (**Table 4**). The largest between-model difference occurred in accuracy (Kendall’s W = 0.346), followed by coherence (W = 0.243) and content accessibility (W = 0.148; **Table 4**; **Figures 4A–C**). **Table 5** presents the Bonferroni-adjusted pairwise comparisons.

**Table 4:** Comparison of five large language models across the five quality dimensions.

Metric	ChatGPT 5.6 Sol	DeepSeek V4 Flash	Qwen 3.8 Max	Kimi K3	Gemini 3.7 Flash	Friedman χ²	Kendall’s W	P
Accuracy	4.67 (4.00–5.00)	3.83 (3.00–4.08)	4.00 (4.00–4.67)	4.00 (3.58–4.33)	3.17 (2.67–4.00)	41.573	0.346	< 0.001
Logic	4.67 (3.92–5.00)	4.00 (4.00–5.00)	4.67 (4.00–5.00)	4.67 (4.00–5.00)	4.00 (4.00–4.42)	5.415	0.045	0.247
Coherence	4.00 (4.00–4.67)	4.17 (4.00–5.00)	3.67 (3.00–4.00)	4.50 (4.00–5.00)	4.00 (4.00–4.42)	29.147	0.243	< 0.001
Safety	4.83 (4.00–5.00)	4.33 (4.00–4.75)	5.00 (4.00–5.00)	4.67 (4.00–5.00)	4.17 (3.67–5.00)	9.010	0.075	0.061
Content accessibility	4.67 (4.00–5.00)	4.33 (4.00–5.00)	4.00 (4.00–4.33)	4.67 (4.00–5.00)	4.00 (3.58–4.08)	17.765	0.148	0.001

Values are presented as median (interquartile range). Overall comparisons used the Friedman test, with Kendall’s W reported as the effect size. Each model contributed 30 responses.

**Figure 4:**
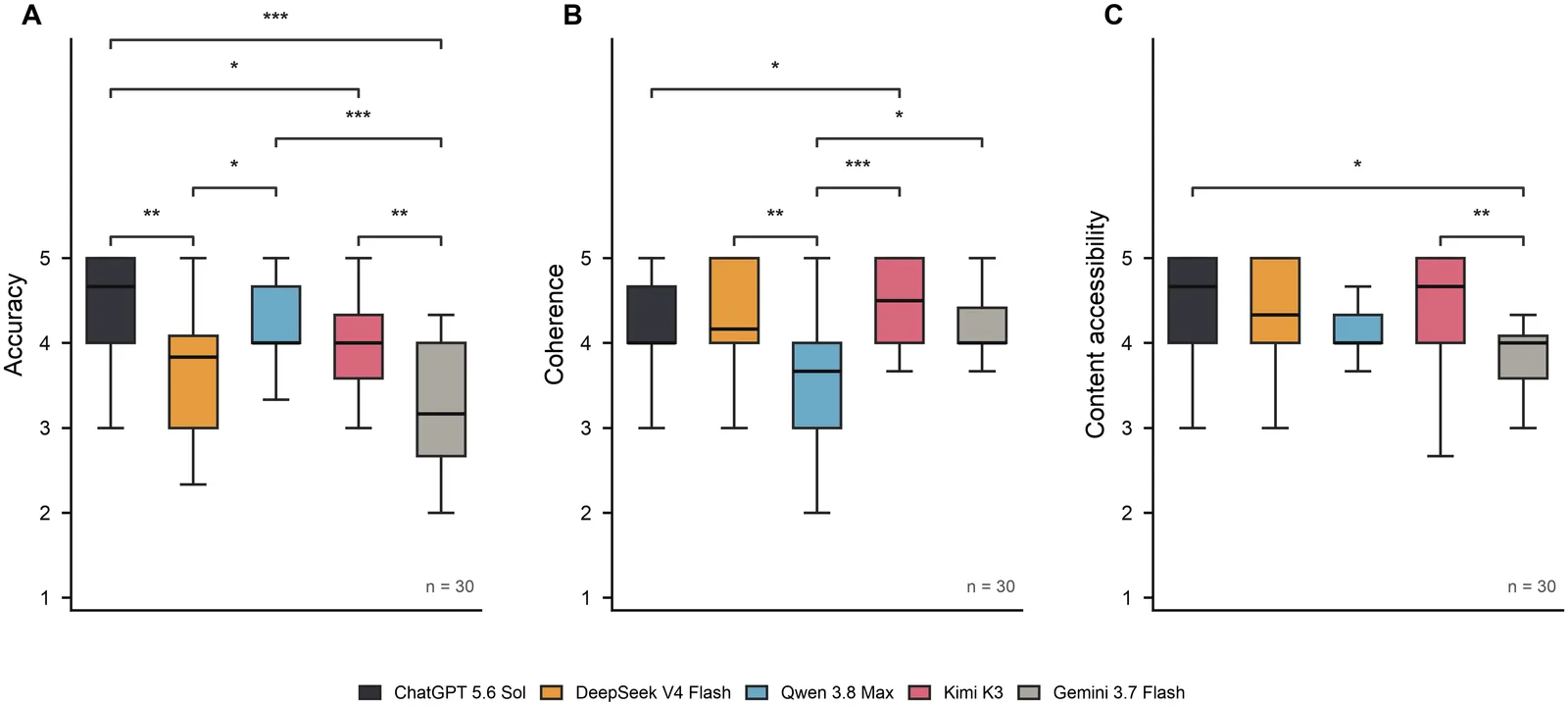
Expert quality ratings for responses from five large language models. Box plots show the distributions of ratings for accuracy **(A)**, coherence **(B)**, and content accessibility **(C)**. Ratings used a 5-point scale, where 1 indicated poor performance and 5 indicated excellent performance. *P < 0.05, **P < 0.01, and ***P < 0.001.

**Table 5:** Pairwise comparisons of expert quality ratings across five large language models.

Comparison	Accuracy	Logic	Coherence	Safety	Content accessibility
ChatGPT 5.6 Sol vs. DeepSeek V4 Flash	0.007	1.000	1.000	1.000	1.000
ChatGPT 5.6 Sol vs. Qwen 3.8 Max	0.531	1.000	0.118	1.000	0.363
ChatGPT 5.6 Sol vs. Kimi K3	0.027	1.000	0.021	1.000	1.000
ChatGPT 5.6 Sol vs. Gemini 3.7 Flash	< 0.001	1.000	1.000	0.682	0.026
DeepSeek V4 Flash vs. Qwen 3.8 Max	0.021	1.000	0.004	0.172	1.000
DeepSeek V4 Flash vs. Kimi K3	1.000	0.847	1.000	0.930	1.000
DeepSeek V4 Flash vs. Gemini 3.7 Flash	0.097	1.000	1.000	1.000	0.558
Qwen 3.8 Max vs. Kimi K3	0.068	1.000	< 0.001	1.000	0.093
Qwen 3.8 Max vs. Gemini 3.7 Flash	< 0.001	0.914	0.024	0.267	1.000
Kimi K3 vs. Gemini 3.7 Flash	0.003	1.000	0.134	0.408	0.007

Pairwise comparisons between models used Wilcoxon signed-rank tests. The table reports Bonferroni-adjusted P values; an adjusted P value < 0.05 was considered statistically significant.

Accuracy ratings differed significantly among models (Friedman χ²(4) = 41.573, Kendall’s W = 0.346, P < 0.001; **Table 4**; **Figure 4A**). ChatGPT 5.6 Sol had the highest median rating [4.67 (IQR, 4.00–5.00)], whereas Gemini 3.7 Flash had the lowest [3.17 (2.67–4.00); **Table 4]**. ChatGPT 5.6 Sol scored higher than DeepSeek V4 Flash (adjusted P = 0.007), Kimi K3 (P = 0.027), and Gemini 3.7 Flash (P < 0.001). Qwen 3.8 Max scored higher than DeepSeek V4 Flash (P = 0.021) and Gemini 3.7 Flash (P < 0.001), and Kimi K3 scored higher than Gemini 3.7 Flash (P = 0.003; **Table 5**; **Figure 4A**). No other comparison reached statistical significance (all adjusted P ≥ 0.068; **Table 5**).

No significant between-model difference was observed in logic ratings (Friedman χ²(4) = 5.415, Kendall’s W = 0.045, P = 0.247; **Table 4**). None of the Bonferroni-adjusted pairwise comparisons reached statistical significance (all adjusted P ≥ 0.847; **Table 5**).

Coherence ratings differed significantly among models (Friedman χ²(4) = 29.147, Kendall’s W = 0.243, P < 0.001; **Table 4**; **Figure 4B**). Kimi K3 had the highest median rating [4.50 (IQR, 4.00–5.00)], whereas Qwen 3.8 Max had the lowest [3.67 (3.00–4.00); **Table 4]**. Kimi K3 scored higher than ChatGPT 5.6 Sol (adjusted P = 0.021) and Qwen 3.8 Max (P < 0.001). DeepSeek V4 Flash and Gemini 3.7 Flash also scored higher than Qwen 3.8 Max (P = 0.004 and P = 0.024, respectively; **Table 5**; **Figure 4B**). No other comparison reached statistical significance (all adjusted P ≥ 0.118; **Table 5**).

No significant between-model difference was observed in safety ratings (Friedman χ²(4) = 9.010, Kendall’s W = 0.075, P = 0.061; **Table 4**). None of the Bonferroni-adjusted pairwise comparisons reached statistical significance (all adjusted P ≥ 0.172; **Table 5**). Final safety scores ≤3 were observed in 2/30 ChatGPT 5.6 Sol responses (6.7%), 3/30 DeepSeek V4 Flash responses (10.0%), 2/30 Qwen 3.8 Max responses (6.7%), 0/30 Kimi K3 responses (0.0%), and 3/30 Gemini 3.7 Flash responses (10.0%). Within this group, one DeepSeek V4 Flash response (1/30; 3.3%) and two Gemini 3.7 Flash responses (2/30; 6.7%) had scores ≤2; no responses from the other three models had scores ≤2.

Content accessibility ratings differed significantly among models (Friedman χ²(4) = 17.765, Kendall’s W = 0.148, P = 0.001; **Table 4**; **Figure 4C**). ChatGPT 5.6 Sol and Kimi K3 both had a median rating of 4.67 (IQR, 4.00–5.00 for both; **Table 4**). Both scored higher than Gemini 3.7 Flash (adjusted P = 0.026 and P = 0.007, respectively; **Table 5**; **Figure 4C**). No other comparison reached statistical significance (all adjusted P ≥ 0.093; **Table 5**).

### 3.3 Associations between objective characteristics and expert-rated quality

After pooling the 150 responses across the five models, we used Spearman rank correlations to describe associations among 11 measures. The 55 unique pairwise tests were adjusted using the Benjamini-Hochberg method. As shown in **Figure 5A**, objective measures of response length were strongly correlated. Word count and non-space character count were almost perfectly correlated (ρ = 0.998, q < 0.001; **Figure 5B**). They were also strongly and positively correlated with sentence count (ρ = 0.831 and 0.827, respectively; both q < 0.001; **Figures 5A, B**). Paragraph count showed moderate positive correlations with word, non-space character, and sentence counts (ρ = 0.450–0.665; all q < 0.001). Response time showed weak-to-moderate positive correlations with word, non-space character, paragraph, and sentence counts (ρ = 0.281–0.435; all q ≤ 0.003; **Figures 5A, B**). **Supplementary Table S4** reports the sample size, Spearman ρ, raw P value, BH-FDR-adjusted q value, and significance determination for all 55 pairwise correlations.

**Figure 5:**
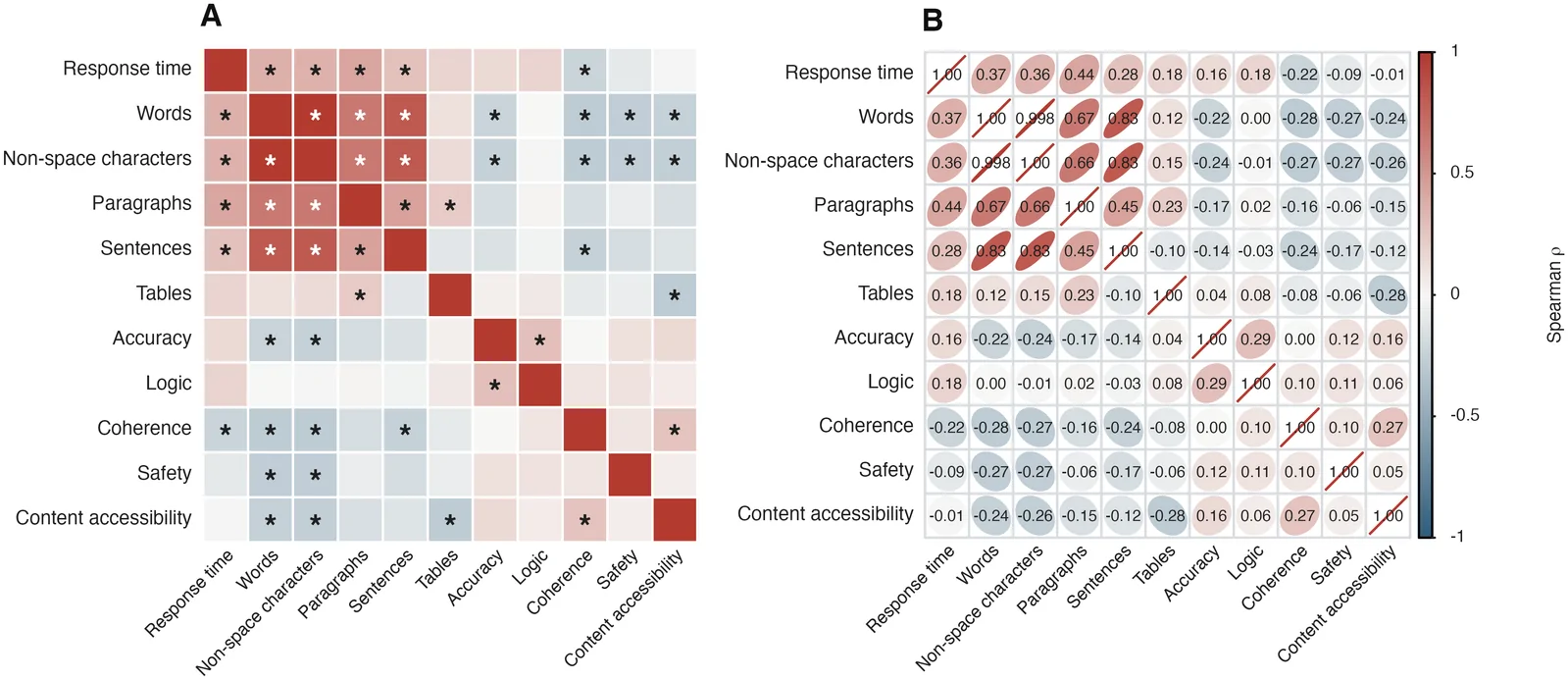
Correlation analysis of objective output characteristics and expert quality ratings. **(A)** Spearman correlation heatmap, with asterisks indicating Benjamini–Hochberg-adjusted q < 0.05. **(B)** Matrix of Spearman correlation coefficients. Red and blue indicate positive and negative correlations, respectively. Color intensity and ellipse shape represent correlation strength. Values are shown to two decimal places, except for the correlation between word count and non-space character count (ρ = 0.998), which is shown to three decimal places.

In contrast, correlations between objective output characteristics and expert ratings were generally weak (|ρ| ≤ 0.279; **Figure 5B**). Word and non-space character counts were weakly negatively correlated with accuracy, coherence, safety, and content accessibility (|ρ| = 0.224–0.276, q = 0.003–0.014; **Figures 5A, B**). Sentence count was weakly negatively correlated with coherence (ρ = −0.241, q = 0.009). Table count was weakly negatively correlated with content accessibility (ρ = −0.279, q = 0.003). Response time was weakly negatively correlated with coherence (ρ = −0.224, q = 0.014; **Figures 5A, B**). After BH-FDR correction, neither the correlation between paragraph count and accuracy (ρ = −0.168, q = 0.078) nor that between response time and accuracy (ρ = 0.158, q = 0.099) reached statistical significance (**Figures 5A, B**).

Correlations among expert-rated dimensions were also weak. Accuracy was weakly positively correlated with logic (ρ = 0.288, q = 0.002), and coherence was weakly positively correlated with content accessibility (ρ = 0.272, q = 0.003; **Figures 5A, B**). The correlation between accuracy and content accessibility was not statistically significant after correction (ρ = 0.160, q = 0.097; **Figures 5A, B**).

## 4 Discussion

Across 30 Chinese-language questions informed by actual clinical inquiries and refined within a guideline-based framework, the five general-purpose LLMs differed in response time, output length, accuracy, coherence, and content accessibility. There was no evidence that any model performed best across all clinically relevant dimensions. ChatGPT 5.6 Sol had relatively high accuracy ratings, Kimi K3 had relatively high coherence ratings, and both performed well in content accessibility. No significant between-model differences were observed in logic or safety. The question bank covered symptom recognition, brain diseases and visual-field changes, care-seeking and testing, treatment, rehabilitation, and psychological support and prognosis. The observed differences therefore reflect more than general information-generation ability. They may affect whether patients understand risks related to central causes, recognize warning signs, and receive appropriate guidance on seeking care. Response time, response length, and text structure also differed among models. However, longer or more structurally complex responses were not necessarily associated with higher expert ratings. Overall, LLM performance in this setting was distinctly multidimensional. Model selection should not rely on a single measure but should consider medical accuracy, content accessibility, and clinical safety together (Bedi et al., 2025).

Different models showed relative strengths in accuracy, coherence, and content accessibility, and correlations among the expert-rated dimensions were generally weak. Correct medical information is a prerequisite for a useful answer in this setting. However, models must also explain central causes, warning signs, timing of care, and diagnostic pathways in language that patients can understand. Previous research found that LLM responses generally performed well in clarity and accessibility but substantially less well in accuracy and completeness. Some responses also contained potentially harmful information (Yau et al., 2024). Similar risks were reported in a study of 100 questions about ocular surface disease. Although ChatGPT-4 performed best among five LLMs, only 59% of questions remained consistently correct across repeated tests, while 28% produced incorrect or confusing responses (Ling et al., 2025). Conversely, a blinded bilingual refractive surgery study illustrates why accuracy and readability require separate assessment. OpenAI o1 received high accuracy and comprehensiveness ratings but had the poorest Chinese readability score among the six models (Tsai et al., 2026). The study’s automated readability measures differ from our expert ratings of content accessibility. Neither approach directly establishes whether patients understand the information or act appropriately on it. The differences observed here may relate to default response styles, optimization for Chinese, and sensitivity to prompting. They cannot yet be attributed entirely to intrinsic model capabilities. Most importantly, no single dimension can replace a multidimensional assessment of whether the information is correct, understandable to patients, and safe. This is particularly important for inquiries that may indicate stroke, an intracranial space-occupying lesion, or another central visual pathway disorder.

Response length likewise could not serve as a substitute for response quality. Word and non-space character counts were almost perfectly correlated (Spearman ρ = 0.998), and both represented the length of the generated content. The differences between mean- and median-based rankings highlight the importance of considering the distributions of response length across questions when comparing models. However, both measures showed weak negative correlations with accuracy, coherence, safety, and content accessibility. Associations between response time and quality ratings were also generally weak and varied in direction. After correction, only response time and coherence showed a weak negative correlation (ρ = −0.224, q = 0.014). The weak positive correlation between response time and accuracy was not statistically significant (ρ = 0.158, q = 0.099). These findings do not imply that longer responses or slower generation reduce quality. In an expert-rated LLM benchmarking study, longer responses generally received higher ratings, and adjusting for response length changed the relative ratings of models (Chen et al., 2026). Another blinded study found that the rating difference between LLM and therapist responses disappeared after response length was controlled. Length itself was a significant predictor of ratings (Shusterman et al., 2025). Thus, response length may influence how raters judge model quality. Another study found that longer physician responses generally received higher ratings for quality and empathy, although their overall ratings remained below those of ChatGPT (GPT-3.5) responses (Ayers et al., 2023). Response length may therefore reflect either redundancy or perceived completeness and explanatory detail. Its effect depends on the task and evaluation criteria.

The absence of a statistically significant difference in safety among the five models, which all received relatively high ratings, does not establish equivalent real-world safety. Median ratings ranged from 4.17 to 5.00 (Friedman χ²(4) = 9.010, P = 0.061). The additional counts showed that some responses received low safety scores despite the high median ratings. With only 30 responses per model, rare serious errors may remain undetected, even when no low-scoring responses are observed. This limitation is particularly relevant because visual field defects associated with brain diseases may require timely stroke assessment or evaluation for intracranial space-occupying lesions. These findings therefore do not support providing medical advice to patients without clinical supervision. A physician-led red-teaming study found that approximately 5%–13.5% of responses across models were rated unsafe by physicians, while 21.6%–43.2% of responses were considered problematic (Draelos et al., 2026). Another study comparing model responses with American Academy of Ophthalmology guidelines reported concordance rates of 81.0%–92.5%. Nevertheless, 1.4%–4.1% of responses were considered potentially harmful (Taloni et al., 2025). The more precise interpretation is that the five models received relatively high expert safety ratings under the present controlled conditions, with no observed between-model difference. Safe patient-facing deployment would still require additional patient information and stronger clinical oversight.

Compared with previous comparative studies, this study focused on a specific Chinese-language setting in which visual field defects were the presenting concern and brain diseases were the principal etiologic context. Bernstein et al. used 200 authentic patient-submitted ophthalmic questions and eight ophthalmologists to compare ChatGPT 3.5 and ophthalmologist responses under blinded conditions. Their study provided an important benchmark for accuracy, appropriateness, and potential harm in real-world patient inquiries, but evaluated only one model and English-language responses (Bernstein et al., 2023). Tailor et al. used a randomized, blinded, multicenter design to compare five LLMs, independent expert responses, and expert-edited LLM responses. Their question set was limited to retinal diseases, and the evaluation focused mainly on quality, empathy, and safety (Tailor et al., 2024). Huang et al. compared five models across 31 common patient-initiated ophthalmology questions. They evaluated accuracy, logic, coherence, safety, and content accessibility (Huang et al., 2025). These studies established important methodological foundations but primarily addressed general ophthalmic questions or a single ocular disease. In contrast, our framework combined six domains: symptom recognition and self-assessment; brain diseases and visual-field changes; care-seeking and diagnostic testing; treatment and intervention; rehabilitation and daily care; and psychological support and prognosis. We specifically assessed LLM performance in the cross-specialty setting of visual field defects associated with brain diseases. To make model comparisons as consistent as possible, the five models were accessed through a unified API client. Identical question text was used within a prespecified testing window, web search was disabled, and conversation history was cleared. This process reduced the influence of hidden prompts, retrieval behavior, and output-format differences (Vishwanath et al., 2026), thereby improving internal consistency across model comparisons. We also analyzed associations between response time, response length, text structure, and the five expert-rated dimensions. Overall, this was a task-level comparison under relatively uniform conditions, with recognition of risks from central causes as its clinical focus. Its conclusions are specific to this setting and do not constitute a general ranking of the models.

Several limitations should be considered when interpreting these findings. First, although informed by actual patient inquiries and refined by experts, the 30 standardized questions could not capture the full range of wording and complexity encountered in clinical consultations. The question categories were also represented unevenly. Each question was also submitted only once to each model. Consequently, within-model variability across repeated generations could not be estimated, and the stability of the observed between-model differences remains untested. The findings are specific to the responses collected under the tested configurations on August 15, 2026. Future studies should assess repeated generations under documented model versions and decoding settings. Second, model parameters could not be fully standardized. Observed end-to-end response times included differences in APIs, networks, API client software, and manual timing error. They should not be interpreted as intrinsic model reasoning speed. Third, web retrieval and external knowledge augmentation were disabled uniformly to improve the internal consistency of model comparisons. The results therefore primarily reflect responses to standardized patient questions without retrieval augmentation. They cannot be directly generalized to deployments using real-time web search, specialized knowledge bases, or retrieval-augmented generation (RAG). Previous research showed that adding a curated professional guideline database to the same GPT-4o model increased guideline-concordant correct responses from 60% to 87%. It also reduced incorrect responses from 12% to 4% and improved response stability and source reliability (Masanneck et al., 2025). Finally, this study evaluated text-only, single-turn interactions and did not include multimodal inputs such as ocular images, medical histories, or other clinical data. Previous ophthalmic research has combined text with slit-lamp or smartphone-acquired ocular images for diagnosis and specialist triage (Kuang et al., 2025). In diabetic macular edema, prediction of response to anti-vascular endothelial growth factor treatment increasingly combines clinical and demographic data with multimodal imaging, including optical coherence tomography (OCT), optical coherence tomography angiography (OCTA), and fluorescein angiography (FA), to obtain more comprehensive patient assessments (Cao et al., 2025). Given these design boundaries, the conclusions should be limited to standardized, text-only, single-turn inquiries without external retrieval. They should not be generalized to systems using real-time search, specialized knowledge bases, or RAG. They also do not establish the diagnostic or triage performance of multimodal systems integrating ocular images and clinical data.

The findings support using LLMs for patient education and clinician-patient communication about visual field defects, rather than allowing them to make diagnostic or treatment decisions independently. Randomized controlled trials in other clinical settings have shown that clinician-reviewed, patient-oriented discharge summaries generated by LLMs can improve patient engagement. Patient-facing chatbots embedded in specialist referral pathways may also shorten consultation time and improve the communication experience (Rust et al., 2026; Tao et al., 2026). Although these studies did not address visual field defects associated with brain diseases, they suggest that LLMs can perform some standardized communication tasks when the intended use is clearly defined and clinical review is retained. Another study found that ChatGPT-4 performed similarly to ophthalmologists without retinal subspecialty training but remained inferior to retinal specialists. It could not replace professional ophthalmic judgment (Wu et al., 2025a). Real-world deployment therefore requires task-specific model selection and careful review of the timeliness, accuracy, and safety of diagnostic and treatment information. Content accessibility must also be considered in patient education. Notably, a randomized controlled trial found that even a clinician-reviewed chatbot with high usability did not significantly improve knowledge gain, anxiety, or satisfaction (Cardon de Lichtbuer et al., 2026). Expert ratings and usability therefore cannot be equated directly with clinical benefit. Clinical review is necessary but not sufficient, and patient-relevant outcomes require prospective validation in clearly defined use cases.

Future research could extend this work in three areas: technical development, multidisciplinary clinical integration, and governance and implementation. Technically, studies could incorporate multi-turn dialogue, retrieval augmentation, and multimodal inputs. They could assess how models answer questions about visual field defects associated with brain diseases when integrating medical histories, visual field plots, OCT data, and neuroimaging reports. Greater emphasis should be placed on identifying rare but high-risk errors. Clinically, prospective studies could be conducted within real care pathways involving ophthalmology or neuro-ophthalmology, neurology, neurosurgery, and visual rehabilitation. Outcomes could include patient understanding, care-seeking decisions, follow-up adherence, anxiety, satisfaction, and clinical outcomes. Governance and implementation research could develop tiered review, human takeover, safety monitoring, and accountability mechanisms. These measures could provide an actionable pathway for regulated use in clinical settings. With such development, LLMs could progress from static response tools toward clinical support systems that can be validated, audited, and overseen, for multidisciplinary patient communication.

## 5 Conclusion

Using a unified API client, fixed questions, single-turn sessions, and no web retrieval, this study compared five leading LLMs: ChatGPT 5.6 Sol, DeepSeek V4 Flash, Gemini 3.7 Flash, Qwen 3.8 Max, and Kimi K3. Each model answered 30 Chinese-language patient questions about visual field defects associated with brain diseases. Performance varied across dimensions, and no model consistently performed best across all clinically relevant domains. Response speed, length, and structure could not replace direct assessment of medical quality. ChatGPT 5.6 Sol received relatively high accuracy ratings, Kimi K3 received relatively high coherence ratings, and both received high understandability ratings. Response length and structural characteristics were only weakly correlated with expert quality ratings and could not be used to infer response quality. The lack of an observed difference in logic or safety under controlled conditions did not establish equivalent or adequate clinical safety. Model selection should therefore be task specific. LLMs should be restricted to patient education and clinician-patient communication under clinical supervision, with particular review of warning signs, timing of care, and cross-specialty guidance. They should not make diagnostic or treatment decisions independently. Future studies should test multi-turn and retrieval-augmented systems, quantify rare but high-risk errors, and evaluate models using patient-relevant outcomes. Such studies are needed to determine whether differences in expert ratings translate into actual clinical benefit.

## Data Availability

The original contributions presented in the study are included in the article/**Supplementary Material**. Further inquiries can be directed to the corresponding authors.
