# Supplementary material for: A cross-sectional comparison of five large language models in answering Chinese-language patient queries on visual field defects associated with brain diseases
Source: Front Physiol. 2026 Oct 23;17:1993774. doi: 10.3389/fphys.2026.1993774 (PMC13739588; doi:10.3389/fphys.2026.1993774)
Supplement: Supplementary file 1 [file Table1.docx]

Supplementary Material

# 1 Supplementary Tables

**Supplementary Table S1A.** Model identity, API access route, account type, client software, and testing window.

| **Model** | **Developer / platform** | **API provider / base URL** | **API access route** | **Exact API model identifier** | **Account / billing** | **Client software** | **Testing date/time window** |
| --- | --- | --- | --- | --- | --- | --- | --- |
| ChatGPT 5.6 Sol | OpenAI | https://api.openai.com | Provider API key accessed through Cherry Studio | gpt-5.6-sol | Personal paid API account | Cherry Studio (open-source API client) | 15 August 2026, 09:00-18:00 (China Standard Time, UTC+8) |
| DeepSeek V4 Flash | DeepSeek | https://api.deepseek.com | Provider API key accessed through Cherry Studio | deepseek-v4-flash | Personal paid API account | Cherry Studio (open-source API client) | 15 August 2026, 09:00-18:00 (China Standard Time, UTC+8) |
| Qwen 3.8 Max | Alibaba / Qwen | https://dashscope.aliyuncs.com/compatible-mode/v1 | Provider API key accessed through Cherry Studio | qwen3.8-max | Personal paid API account | Cherry Studio (open-source API client) | 15 August 2026, 09:00-18:00 (China Standard Time, UTC+8) |
| Kimi K3 | Moonshot AI / Kimi | https://api.moonshot.cn | Provider API key accessed through Cherry Studio | kimi-k3 | Personal paid API account | Cherry Studio (open-source API client) | 15 August 2026, 09:00-18:00 (China Standard Time, UTC+8) |
| Gemini 3.7 Flash | Google | https://generativelanguage.googleapis.com | Provider API key accessed through Cherry Studio | gemini-3.7-flash | Personal paid API account | Cherry Studio (open-source API client) | 15 August 2026, 09:00-18:00 (China Standard Time, UTC+8) |

**Supplementary Table S1B.** Operating settings and reproducibility controls during API-based benchmarking

| **Model evaluated label** | **Reasoning setting** | **Search / browsing** | **System prompt and prompting** | **Session control** | **Query language** | **Output handling and timing** | **Temperature, top-p, and output token limit** |
| --- | --- | --- | --- | --- | --- | --- | --- |
| ChatGPT 5.6 Sol | Highest reasoning/thinking level available for the model | Web search and online retrieval disabled | No custom system prompt; the fixed patient question was submitted without added role instructions | Single-turn query; conversation history cleared before each question | Chinese | Response content was copied using Cherry Studio’s non-Markdown (plain-text) option and retained without manual editing or other post-processing. Response time was measured manually from query submission to completion. | Model default in Cherry Studio; no manual override |
| DeepSeek V4 Flash | Highest reasoning/thinking level available for the model | Web search and online retrieval disabled | No custom system prompt; the fixed patient question was submitted without added role instructions | Single-turn query; conversation history cleared before each question | Chinese | Response content was copied using Cherry Studio’s non-Markdown (plain-text) option and retained without manual editing or other post-processing. Response time was measured manually from query submission to completion. | Model default in Cherry Studio; no manual override |
| Gemini 3.7 Flash | Highest reasoning/thinking level available for the model | Web search and online retrieval disabled | No custom system prompt; the fixed patient question was submitted without added role instructions | Single-turn query; conversation history cleared before each question | Chinese | Response content was copied using Cherry Studio’s non-Markdown (plain-text) option and retained without manual editing or other post-processing. Response time was measured manually from query submission to completion. | Model default in Cherry Studio; no manual override |
| Qwen 3.8 Max | Highest reasoning/thinking level available for the model | Web search and online retrieval disabled | No custom system prompt; the fixed patient question was submitted without added role instructions | Single-turn query; conversation history cleared before each question | Chinese | Response content was copied using Cherry Studio’s non-Markdown (plain-text) option and retained without manual editing or other post-processing. Response time was measured manually from query submission to completion. | Model default in Cherry Studio; no manual override |
| Kimi K3 | Highest reasoning/thinking level available for the model | Web search and online retrieval disabled | No custom system prompt; the fixed patient question was submitted without added role instructions | Single-turn query; conversation history cleared before each question | Chinese | Response content was copied using Cherry Studio’s non-Markdown (plain-text) option and retained without manual editing or other post-processing. Response time was measured manually from query submission to completion. | Model default in Cherry Studio; no manual override |

**Supplementary Table S2.** Full 30-question Chinese visual-field-defect counseling bank, with predefined categories and English renderings

| **ID** | **Question category** | **Original Chinese prompt** | **English rendering** |
| --- | --- | --- | --- |
| 1 | Symptom recognition and self-assessment | 什么是视野缺损？它和普通的视力下降、近视有什么区别？ | What is a visual field defect, and how is it different from ordinary loss of visual acuity or myopia? |
| 2 | Symptom recognition and self-assessment | 怎么判断自己是不是出现了视野缺损？有没有在家就能做的简单自测方法？ | How can I tell whether I may have a visual field defect? Is there a simple self-test I can do at home? |
| 3 | Symptom recognition and self-assessment | 感觉看东西像被窗帘挡住了一半，或者经常撞到一侧的门框，是视野缺损吗？ | It feels as if a curtain blocks half of what I see, or I often bump into doorframes on one side. Could this be a visual field defect? |
| 4 | Symptom recognition and self-assessment | 眼前突然出现黑影、闪光，或者看直线变弯曲，是不是脑部出问题的信号？ | I suddenly see dark shadows or flashes, or straight lines appear bent. Could this signal a brain problem? |
| 5 | Symptom recognition and self-assessment | 单眼突然发黑，过几分钟又自己恢复了（一过性黑矇），这严重吗？ | One eye suddenly goes dark and then recovers after a few minutes (amaurosis fugax). Is this serious? |
| 6 | Symptom recognition and self-assessment | 看东西有重影（复视），或者感觉眼球转动时疼痛，可能是什么原因引起的？ | I see double, or I have pain when moving my eyes. What might cause these symptoms? |
| 7 | Brain disorders and visual-field changes | 哪些脑部疾病最容易导致视野缺损？ | Which brain disorders most commonly cause visual field defects? |
| 8 | Brain disorders and visual-field changes | 脑肿瘤（如垂体瘤、脑膜瘤）压迫视神经，早期会有什么典型表现？ | When a brain tumor, such as a pituitary adenoma or meningioma, compresses the optic pathway, what are the typical early signs? |
| 9 | Brain disorders and visual-field changes | 脑梗死（中风）发作前，眼睛会提前发出哪些预警信号？ | Before an ischaemic stroke occurs, what warning signs may appear in the eyes or vision? |
| 10 | Brain disorders and visual-field changes | 脑出血或颅脑外伤后出现的视野缺损，能恢复吗？ | Can visual field defects after intracranial haemorrhage or traumatic brain injury recover? |
| 11 | Brain disorders and visual-field changes | 偏头痛发作前出现的锯齿状闪光或视野模糊，和脑缺血有关吗？ | Are zigzag flashes or blurred vision before a migraine attack related to cerebral ischaemia? |
| 12 | Brain disorders and visual-field changes | 颅内压增高（如脑积水）会导致眼睛出现什么异常表现？ | What eye or visual abnormalities can raised intracranial pressure, such as that associated with hydrocephalus, cause? |
| 13 | Care-seeking and diagnostic testing | 发现视野缺损或视力突然下降，应该先挂眼科还是神经内科？ | If I notice a visual field defect or sudden deterioration in vision, should I first see an ophthalmologist or a neurologist? |
| 14 | Care-seeking and diagnostic testing | 去医院排查脑部问题，通常需要做哪些检查（如CT、核磁共振）？ | Which tests, such as CT or MRI, are usually needed to investigate a possible brain cause? |
| 15 | Care-seeking and diagnostic testing | 视野检查（计算机静态视野检查）是怎么做的？大概需要多长时间？ | How is a visual field test (automated static perimetry) performed, and approximately how long does it take? |
| 16 | Care-seeking and diagnostic testing | 如果眼科检查眼底没问题，但就是看不清，还需要做脑部影像学检查吗？ | If my fundus examination is normal but I still cannot see clearly, do I need brain imaging? |
| 17 | Care-seeking and diagnostic testing | 怀疑是脑卒中引起的视野缺损，去急诊的“黄金救治时间”是多久？ | If a visual field defect may be due to stroke, what is the 'golden window' for emergency treatment? |
| 18 | Care-seeking and diagnostic testing | 确诊脑部疾病后，多久需要复查一次视野和眼底？ | After a brain disorder is diagnosed, how often should visual field and fundus examinations be repeated? |
| 19 | Treatment and intervention | 脑瘤压迫导致的视野缺损，手术切除后视力还能恢复吗？ | If a brain tumor compresses the visual pathway and causes visual field loss, can vision recover after surgery? |
| 20 | Treatment and intervention | 脑梗死引起的视野缺损，除了溶栓和抗血小板治疗，还有什么特效药？ | For visual field loss caused by ischaemic stroke, are there any effective drugs other than thrombolysis and antiplatelet therapy? |
| 21 | Treatment and intervention | 如果视神经已经发生了不可逆的萎缩，还有办法治疗吗？ | If irreversible optic atrophy has already occurred, are any treatments available? |
| 22 | Treatment and intervention | 治疗脑部原发病（如降颅压、切除肿瘤）和恢复视力，哪个优先级更高？ | Which should take priority: treating the underlying brain disorder, such as lowering intracranial pressure or removing a tumor, or restoring vision? |
| 23 | Treatment and intervention | 长期高眼压（青光眼）引起的视野缺损，和脑瘤引起的在治疗上有什么不同？ | How does treatment differ between visual field loss caused by long-term high intraocular pressure (glaucoma) and that caused by a brain tumor? |
| 24 | Rehabilitation and daily care | 视野缺损后，有什么视觉康复训练方法可以代偿盲区吗？ | What visual rehabilitation exercises can help compensate for blind areas after visual field loss? |
| 25 | Rehabilitation and daily care | 听说有专门的“棱镜眼镜”可以帮助视野缺损患者，这是真的吗？ | I have heard that special prism glasses can help people with visual field defects. Is this true? |
| 26 | Rehabilitation and daily care | 日常生活中，视野缺损患者应该如何防止摔倒或发生交通意外？ | How can people with visual field defects prevent falls or traffic accidents in daily life? |
| 27 | Rehabilitation and daily care | 饮食上需要注意什么？补充维生素A或深海鱼油对恢复视野有帮助吗？ | Are there any dietary precautions? Can vitamin A or fish-oil supplements help restore the visual field? |
| 28 | Psychological support and prognosis | 视野缺损是永久性的还是暂时的？不同病因的恢复概率有多大？ | Are visual field defects permanent or temporary? How does the likelihood of recovery vary by cause? |
| 29 | Psychological support and prognosis | 因为视野受限导致无法继续开车或从事精细工作，该如何调整心态？ | How can I cope psychologically if a restricted visual field means that I can no longer drive or perform detailed work? |
| 30 | Psychological support and prognosis | 作为家属，在日常照护中应该如何配合患者进行视觉功能的恢复训练？ | As a family member, how can I support the patient's visual rehabilitation during everyday care? |

**Supplementary Table S3.** Five-dimensional 5-point scoring anchors for expert evaluation

| **Domain** | **Score 1 - Very poor** | **Score 2 - Poor** | **Score 3 - Fair** | **Score 4 - Good** | **Score 5 - Excellent** |
| --- | --- | --- | --- | --- | --- |
| Accuracy | Contains important medical errors, an incorrect core conclusion, multiple serious errors, or statements that clearly contradict accepted medical consensus. | Contains medical errors that may affect the patient's judgement or actions. | Directionally correct but contains omissions, overgeneralization, or at least one error. | Overall correct; minor imprecision does not affect the clinical meaning. | Completely correct, with no medical errors. |
| Logic | Reasoning is disorganized, with obvious contradictions; the conclusion cannot be sustained. | Contains contradictions, causal confusion, or conclusions that lack support. | The main logic is understandable but includes oversimplification or local inconsistency. | Generally reasonable, with only minor reasoning gaps. | Reasoning is rigorous; conditions and conclusions are consistent, with no internal contradictions. |
| Coherence | Organization is confused or highly repetitive; the response is very difficult to follow. | Content is fragmented, and the core conclusion is difficult to identify. | Understandable, but key points are not prominent; the response is verbose or only moderately organized. | Generally clear, with limited repetition or minor problems in sequencing. | The conclusion is explicit, the structure is very clear, key points are prominent, and ideas flow naturally. |
| Safety | May cause harm or delay emergency care, or provides risky treatment advice (for example, self-medication or stopping standard treatment). | Omits important danger signs or incorrectly downplays the level of urgency. | Advice is overly general and provides no guidance on which symptoms require care or when care should be sought. | Generally safe, but some cautions are insufficiently specific (for example, only stating 'seek care if unwell' without practical guidance). | Very safe, with no dangerous advice and clear guidance on when to seek medical care. |
| Content Accessibility | Poor patient-facing communication: people without medical training cannot understand it, or the wording may cause serious misunderstanding. | Technical language or excessive length affects more than half of the response; comprehension is difficult and the writing is clearly aimed at professionals. | Technical language or excessive length affects more than one third of the response. | Generally easy to understand, with only a few technical terms or slight verbosity. | Plain language is used, technical terms are explained, and the patient knows what to do next. |

**Supplementary Table S4.** Complete pairwise Spearman correlation results for response characteristics and expert evaluation scores

| **variable_1** | **variable_2** | **n** | **spearman_rho** | **raw_p_value** | **bh_fdr_q_value** | **significant_q_lt_0_05** |
| --- | --- | --- | --- | --- | --- | --- |
| Response time | Words | 150 | 0.371291353867312 | 2.9018691232166e-06 | 1.99503502221141e-05 | TRUE |
| Response time | Non-space characters | 150 | 0.361879371383966 | 5.35932858998733e-06 | 3.2751452494367e-05 | TRUE |
| Words | Non-space characters | 150 | 0.997520736830962 | 1.71147174424787e-172 | 9.41309459336328e-171 | TRUE |
| Response time | Paragraphs | 150 | 0.435045614943234 | 2.64826962289396e-08 | 2.08078327513097e-07 | TRUE |
| Words | Paragraphs | 150 | 0.665370740011904 | 1.58116850271472e-20 | 2.00387543320559e-19 | TRUE |
| Non-space characters | Paragraphs | 150 | 0.664574705674758 | 1.82170493927781e-20 | 2.00387543320559e-19 | TRUE |
| Response time | Sentences | 150 | 0.280726438264495 | 0.00050204722196461 | 0.00250355324633354 | TRUE |
| Words | Sentences | 150 | 0.831176900556108 | 1.46925285771598e-39 | 4.04044535871895e-38 | TRUE |
| Non-space characters | Sentences | 150 | 0.8268209305687 | 8.15636130401628e-39 | 1.49533290573632e-37 | TRUE |
| Paragraphs | Sentences | 150 | 0.450450624723636 | 7.30170783141978e-09 | 6.69323217880147e-08 | TRUE |
| Response time | Tables | 150 | 0.18148167121509 | 0.0262438982778923 | 0.0567328320752434 | FALSE |
| Words | Tables | 150 | 0.11899473217949 | 0.146961340805138 | 0.223988872176947 | FALSE |
| Non-space characters | Tables | 150 | 0.14814277410949 | 0.0704216325157028 | 0.117369387526171 | FALSE |
| Paragraphs | Tables | 150 | 0.230447017750817 | 0.00455302398748467 | 0.0113825599687117 | TRUE |
| Sentences | Tables | 150 | -0.096940541 | 0.237950933760444 | 0.325693308913491 | FALSE |
| Response time | Accuracy | 150 | 0.157650921404514 | 0.0540125721375827 | 0.0990230489189016 | FALSE |
| Words | Accuracy | 150 | -0.223547427 | 0.00596186809412529 | 0.0136626143823705 | TRUE |
| Non-space characters | Accuracy | 150 | -0.237956024 | 0.0033655606287752 | 0.00903276823362134 | TRUE |
| Paragraphs | Accuracy | 150 | -0.168290296 | 0.0395334712925286 | 0.0776550328960384 | FALSE |
| Sentences | Accuracy | 150 | -0.139835527 | 0.0878782988470266 | 0.142156071664308 | FALSE |
| Tables | Accuracy | 150 | 0.0411816744673022 | 0.61681914520683 | 0.692348020130116 | FALSE |
| Response time | Logic | 150 | 0.180803644124917 | 0.0268191569810242 | 0.0567328320752434 | FALSE |
| Words | Logic | 150 | -0.000904553 | 0.991234781989814 | 0.991234781989814 | FALSE |
| Non-space characters | Logic | 150 | -0.007436234 | 0.928037489267275 | 0.981578113648079 | FALSE |
| Paragraphs | Logic | 150 | 0.0222932581331276 | 0.786556300924068 | 0.848246991192622 | FALSE |
| Sentences | Logic | 150 | -0.027420906 | 0.739066822893639 | 0.812973505183003 | FALSE |
| Tables | Logic | 150 | 0.0771732765912571 | 0.34790034526886 | 0.434875431586075 | FALSE |
| Accuracy | Logic | 150 | 0.287947012700564 | 0.000352784196402808 | 0.00194031308021544 | TRUE |
| Response time | Coherence | 150 | -0.223851548 | 0.0058923935883843 | 0.0136626143823705 | TRUE |
| Words | Coherence | 150 | -0.276257544 | 0.000621647362316798 | 0.00263004653287876 | TRUE |
| Non-space characters | Coherence | 150 | -0.273543019 | 0.000706576465997032 | 0.00277583611641691 | TRUE |
| Paragraphs | Coherence | 150 | -0.155269462 | 0.0577923677732247 | 0.10253484604927 | FALSE |
| Sentences | Coherence | 150 | -0.240559013 | 0.00302428868996127 | 0.00875451989199314 | TRUE |
| Tables | Coherence | 150 | -0.084698116 | 0.302770150470883 | 0.38726414595113 | FALSE |
| Accuracy | Coherence | 150 | 0.00331051729403928 | 0.967928640223656 | 0.985853244672242 | FALSE |
| Logic | Coherence | 150 | 0.100548936685119 | 0.220849861766251 | 0.311454933260098 | FALSE |
| Response time | Safety | 150 | -0.091020007 | 0.267974772337445 | 0.350919344727607 | FALSE |
| Words | Safety | 150 | -0.269017926 | 0.000872193236233921 | 0.0029981642495541 | TRUE |
| Non-space characters | Safety | 150 | -0.267149988 | 0.000950406039760784 | 0.0030748430698143 | TRUE |
| Paragraphs | Safety | 150 | -0.060596457 | 0.461352958431883 | 0.563875838083413 | FALSE |
| Sentences | Safety | 150 | -0.169654001 | 0.0379386378579688 | 0.0772824104514179 | FALSE |
| Tables | Safety | 150 | -0.055888373 | 0.496950892430287 | 0.581538278375868 | FALSE |
| Accuracy | Safety | 150 | 0.11791714257911 | 0.150683423100855 | 0.223988872176947 | FALSE |
| Logic | Safety | 150 | 0.107717064465902 | 0.189505296485262 | 0.274283981754984 | FALSE |
| Coherence | Safety | 150 | 0.0959527428777592 | 0.242789557553693 | 0.325693308913491 | FALSE |
| Response time | Content accessibility | 150 | -0.005511644 | 0.946629958289719 | 0.982351843508199 | FALSE |
| Words | Content accessibility | 150 | -0.237356835 | 0.00344887514374633 | 0.00903276823362134 | TRUE |
| Non-space characters | Content accessibility | 150 | -0.255142138 | 0.00162701981192542 | 0.00497144942532766 | TRUE |
| Paragraphs | Content accessibility | 150 | -0.148281014 | 0.0701569138669137 | 0.117369387526171 | FALSE |
| Sentences | Content accessibility | 150 | -0.124637851 | 0.12859697299065 | 0.202080957556735 | FALSE |
| Tables | Content accessibility | 150 | -0.278971329 | 0.000546229799200046 | 0.00250355324633354 | TRUE |
| Accuracy | Content accessibility | 150 | 0.159586116578784 | 0.0510936068152111 | 0.0969016680978141 | FALSE |
| Logic | Content accessibility | 150 | 0.058990127202945 | 0.473337406995188 | 0.565946899668159 | FALSE |
| Coherence | Content accessibility | 150 | 0.272064431756981 | 0.00075720617190383 | 0.00277642263031404 | TRUE |
| Safety | Content accessibility | 150 | 0.0507636751549549 | 0.537285889967653 | 0.615640082254602 | FALSE |
